# Association of VNTR polymorphism of tumor necrosis factor receptor 2 (*TNFRSF1B*) with pulmonary tuberculosis

**Published:** 2017-03

**Authors:** Mohammad Naderi, Mohammad Hashemi, Nazanin Moradi

**Affiliations:** 1 Infectious Diseases and Tropical Medicine Research Center, Zahedan University of Medical Sciences, Zahedan, Iran; 2 Department of Clinical Biochemistry, School of Medicine, Zahedan University of Medical Sciences, Zahedan, Iran

**Keywords:** Pulmonary tuberculosis, *TNFRSF1B*, *VNTR*, Polymorphism

## Abstract

This study was designed to find out the impact of the variable number of tandem repeats (VNTR) of the tumor necrosis factor receptor 2 (*TNFRSF1B*) on pulmonary tuberculosis (PTB) risk in an Iranian population. This case-control study was done on 159 PTB patients and 158 healthy subjects. Bi-allelic *TNFRSF1B* VNTR was genotyped by polymerase chain reaction. Logistic regression analysis revealed no significant association between *TNFRSF1B* VNTR and PTB risk (P>0.05). Our findings proposed that *TNFRSF1B* VNTR polymorphism is unlikely to confer susceptibility to PTB.

## INTRODUCTION

Tuberculosis (TB), caused by *Mycobacterium tuberculosis*, is one of the major public health problem worldwide. In 2015, according to the global tuberculosis report from WHO, there were an estimated 10.4 million new TB cases and 1.4 million TB deaths worldwide [1]. One-third of the world’s population is supposed to be infected with *Mycobacterium tuberculosis*, while approximately 10% of infected cases will develop active TB disease during their lifetimes [2]. It has been proposed that host genetic variations play a key role in determining differential vulnerability to TB [3-6].

Tumor necrosis factor (TNF), a pleiotropic cytokine, plays a key role in mediating various immune functions including inflammation [7, 8], the regulation of apoptosis and necrosis [3], and induction of cytotoxicity [4]. TNF-α is capable of eliciting a variety of different immune responses by signaling via two types of membrane-bound receptors, TNFR1 (CD120a, TNFRSF1A) and TNFR2 (CD120b, TNFRSF1B, OMIM: 191191) [5, 6]. TNFR1 expressed on all cell types while TNFR2 expressed mainly on cells of the immune system [6, 7]. The immune response to *M.* *tuberculosis* infection seems to be a balance between inflammatory and anti-inflammatory responses in which TNF-α plays a key role [9]. It has been proposed that TNF-α is a macrophage-activating cytokine that stimulates phagocytosis, anti-mycobacterial mechanism in macrophage and is a potent inducer of apoptosis [10]. Some variants of the *TNFRSF1B* polymorphisms could affect the receptor activity and they have been considered as possible markers of host susceptibility to TB [9, 11]. To the best of our knowledge, no studies have yet inspected the possible association between the* TNFRSF1B* VNTR variant and susceptibility to PTB infection. Thus, the present study was carried out.

## MATERIALS AND METHODS

We performed a case-control study with 159 (58 men and 101 women; age 50.8± 20.3 years) confirmed PTB and 158 (68 men and 90 women; age 47.2±14.7 years) healthy subjects. The individuals participating in this study were Baluch, Sistani and Persian ethnicities. There was no significant difference between the group regarding gender (P=0.252) and age (P=0.073). The cases were chosen from PTB patients admitted to a university-affiliated hospital (Bou-Ali Hospital, Zahedan, referral center for TB). The study design and the enrollment procedure have been defined in our previous publication [12]. All controls were unrelated adults selected through the population without recent sign, symptom or history of TB and from the same geographical origin, as the patients with PTB. The local ethical research committee of the Zahedan University of Medical Sciences approved the project and informed consent was taken from participants. The genotyping of the *TNFRSF1B* VNTR was done as described previously [13]. Data were analyzed using t-test and χ^2^ According to the data using SPSS 22 software. The odds ratio (OR) and 95% confidence interval (CI) were estimated from unconditional logistic regression analyses. A P value <0.05 was considered as statistically significant.

## RESULTS AND DISCUSSION

The genotype and allele frequencies of *TNFRSF1B* VNTR are shown in Table 1. The *TNFRSF1B* genotypes in controls were in Hardy-Weinberg equilibrium (χ^2^=0.106, df=1 P=0.747). The results showed that *TNFRSF1B* VNTR polymorphism was not associated with the risk of PTB in the codominant, dominant, and recessive inheritance models tested. The allele 1 was not associated with risk of PTB compared to allele 2. 

 TNF-α is a pro-inflammatory cytokine that plays a vital role in the development of the immune response. It performs its biological activity via binding to two distinct cell surface receptors TNFR1 and TNFR2, with molecular masses of 55KD and 75KD, respectively) [14, 15]. TNF and its receptors (TNFR1 and TNFR2) are crucial to control M. tuberculosis infection [16]. 

It has been reported that rs3397 T allele and the 3` untranslated region (3`UTR) haplotype GTT (rs1061624 A/G, rs5030792 G/T, rs3397 C/T) was associated with resistance to TB [11]. The possible association between 3`UTR polymorphisms (rs1061624 A/G, rs5030792 G/T, rs3397 G/T) of *TNFRSF1B* and risk of TB in Han Chinese pediatric population was reported [9]. Their findings showed that rs5030792 variant was not polymorphic and all participants in cases and controls were TT genotype. The rs1061624 variant was associated with TB risk, while the findings did not support an association between rs3397 variant and the risk of TB [9].

**Table 1:** The genotypes and allele distribution of *TNFRSF1B* VNTR in pulmonary tuberculosis (PTB) and control groups

***TNFRII*** ** VNTR**	**PTB (%)**	**Control (%)**	**OR **	**95% CI**	**P**
**Codominant**					
**2/2**	89 (56.0)	98 (62.0)	1.00	-	-
**2/1**	56 (35.2)	52 (32.9)	1.19	0.74-1.91	0.546
**1/1**	14 (8.8)	8 (5.1)	1.93	0.77-4.81	0.180
**Dominant**					
**2/2**	89 (56.0)	98 (62.0)	1.00	-	-
**2/1+1/1**	70 (44.0)	60 (38.0)	1.24	0.84-1.82	0.321
**Alleles**					
**2**	234 (73.6)	248 (78.5)	1.00	-	-
**1**	84 (26.4)	68 (21.5)	1.31	0.91-1.89	0.163

The findings of several systems strongly propose that a bi-allelic VNTR in the promoter of the *TNFRSF1B* gene, which involves the 15-bp insertion/deletion, has a role in signal transduction during pathogenesis [17], thus it can play an active role in elicitation of cytokine secretion [18]. It has been shown that the transmembrane form of TNF-α is the main activating ligand of TNFR2 and it can give qualitatively different results in comparison with soluble TNF-α [19]. One of the limitations of the current study is its relatively small sample size. Larger studies among different ethnicities are necessary.
